# A method for estimating magnetic target location by employing total field and its gradients data

**DOI:** 10.1038/s41598-022-22725-9

**Published:** 2022-10-26

**Authors:** Haidong You, Jindong Li, Jun Xu, Jian Xu, Tigang Ning, Yuanyuan Gao, Lin Li

**Affiliations:** 1 Science and Information College, Qingdao Agricultural University, Qingdao, 266109 China; 2 Institute of Lightwave Technology, Beijing Jiaotong University, Beijing, 100044 China; 3 College of Chemistry and Pharmaceutical Sciences, Qingdao Agricultural University, Qingdao, 266109 China

**Keywords:** Techniques and instrumentation, Geomagnetism

## Abstract

In this paper, we present a magnetic target localization method by measurement of total field and its spatial gradients. We deduce an approximate formula of the target’s bearing vector expressed by the total field and its gradients. The total field and its gradient can be measured by a scalar magnetometer array and the approximate value of the bearing vector can be calculated. An iterative method is introduced to improve the localization accuracy of the magnetic target. Simulations experiments have been done to evaluate the performance of the proposed method. The results show that the relative errors of the bearing vector estimated by the iterative method can be kept below the level of 5%. In addition, when difference root-mean-square (RMS) noise is added to the magnetometers, the relative errors of the bearing vector only vary from 0.8 to 6%, which indicates that the proposed method has a high tolerance to the noise of the magnetometers.

## Introduction

The magnetic field generated by a remote magnetic target can be considered as a magnetic dipole^1,2^. Magnetic dipole target location is a valuable way to detect the magnetic target. And so far, several methods for magnetic dipole target location have been previously proposed. Usually, we can measure the origin data by vector magnetometers or scalar magnetometers, afterward, the target location can be estimated by solving nonlinear equations or analytical solutions with the measured data.

A vector magnetometers can provide valuable target data. Three components of the filed induced by the magnetic target can be measured by the vector magnetometers and then magnetic gradient tensor or tensor invariants also can be calculated. The target location can be inverted by either three components, or the gradient tensor, or the tensor invariants, or a combination of them^3–5^. In 2006, Nara and co-workers proposed a simple algorithm for localization of a magnetic dipole by measurement of magnetic field vector and spatial gradients^6^. A magnetic dipole target location scheme was proposed in 2007^7^ which is based on spatial variations of magnetic gradient tensor invariants, this scheme is called as Scalar Triangulation And Ranging (STAR) method. The STAR method assumes that the magnetic gradient tensor invariant is a perfect sphere, which is not completely accurate. Hence, the STAR method always causes localization errors. To correct the asphericity errors of traditional STAR and achieve better location accuracy, some methods have been proposed in recent years. By comparison of the invariants obtained by the initial bearing vector and the sensor group, the asphericity errors can be iteratively reduced^8^. Wang^9^ proposed another iteratively algorithm to improve the STAR method, the main idea is that the unit bearing vector and the magnetic gradient contraction are updated during every step.

Clark^10,11^ firstly defined the Normalized Source Strength (NSS) which obviates the problem that the isosurface of the tensor contraction is an ellipsoid. NSS is a particularly useful rotational invariant and can be calculated from the eigenvalues of the tensor. Yang^12^ provided a closed formula for the estimation of bearing vector by NSS. In this method, NSS is used to replace the old one whose contour is a sphere instead of the ellipsoid. Yin^13^ gived another analytical expression of bearing vector by employing NSS. According to this method, three unknown components of bearing vector are established by solving the equations about the NSS of six faces of the vector magnetometer cube, at the same time, the magnitude of magnetic moment vector also can be obtained. In addition, magnetic dipole localization can be achieved with a closed–form formula by measurement of its magnetic field vector and magnetic gradient tensor^14^. In 2021, Xu^15^ proposed a linear method based on the two-point magnetic gradient full tensor for magnetic diploe location and the principle of single-point magnetic gradient full tensor positioning is analyzed in detail. However, for the above mentioned magnetic dipole target location scheme, the measurement system is consisted by a set of 8 vector magnetometers in a cubic array and 24 channels of magnetic field are measured, which is complexity and the strict alignment for the vector magnetometers in the cubic array is hard. These reasons will affect the location accuracy.

A scalar magnetometer is relatively insensitive to its orientation and is easier to construct the field measurement system than a vector magnetometer. The magnetic diploe location schemes have been verified in recent years with scalar magnetometers. Zalevsky^16^ presented a high resolution automatic detection algorithm based on a scalar magnetometer array. Fan^17^ obtained a scheme for tracking of moving magnetic diploe and the particle swarm optimization (PSO) algorithm is employed to solve the high-order nonlinear equations for estimating the position of the magnetic diploe. But the accuracy of the location is intrinsically limited by the PSO optimization algorithm. Kang^18^ presented a method of locating a magnetic diploe based on a scalar magnetometer array. In the method, a conjugate gradient algorithm was designed and the main idea is that the moving target is measured twice with a time interval, thus, the influence of locating target can be eliminated which is caused by the geomagnetic total field with time-varying and uneven spatial distribution. As same as the Fan’s method^17^, the nonlinear equations are solved by LINGO, which cause the lower locating accuracy.

In this study, we focused on the localization of magnetic diploe by using a scalar magnetometer array. We proposed a method for estimating magnetic target location based on total field and its gradients data. The iterative algorithm is used to improve the positioning accuracy. Simulations have been done to verify the performance of the presented method. The results show that the method of locating target is feasible.

## Results

### Bearing vector of the magnetic target

A magnetic target can be considered as a magnetic diploe when the distance between the target and measurement array is more than 2.5 times the size of target. The magnetic field generated by the magnetic diploe cat be expressed as^19^

1 $${\mathbf{B}} = \frac{{\mu_{0} }}{{4\pi r^{5} }}[3({\mathbf{M}} \cdot {\mathbf{r}}){\mathbf{r}} - r^{2} {\mathbf{M}}]$$

where *μ*_0_ = 4*π* × 10^–7^ H/m is the permeability of free space. **r** = *rr*_**0**_ is the bearing vector. **r**_**0**_ is the unit vector of **r**, *r* is the magnitude of **r**. **M** = *Mm*_**0**_ is the magnetic moment of magnetic diploe. *M* is the magnitude of **M**, **m**_**0**_ is the unit vector of **M**. “**.**” presents the dot product of vectors.

The magnitude of **B** can be calculated as

2 $$\left| {\mathbf{B}} \right| = \frac{\mu Ms}{{4\pi r^{3} }}$$

where *s* = [1 + 3(*t*)^2^]^1/2^ and *t* = **r**_**0**_**⋅m**_**0**_. The magnitude of **B** can be measured by a scalar magnetometer. As can be seen in Eq. (2), |**B**| can be seen as a rotational invariant^20^. |**B**| only includes the magnitude of **r** and bearing information of the magnetic target can’t be obtained. In order to figure out the bearing information of the magnetic target, further, parameter *s* is assumed to be a spatial constant, we calculated the gradient of |**B**| as follows

3 $${\mathbf{G}} = \nabla \left| {\mathbf{B}} \right| = - \frac{3\mu Ms}{{4\pi r^{4} }}{\mathbf{r}}_{{\mathbf{0}}}$$

and the magnitude of **G** is

4 $$\left| {\mathbf{G}} \right| = \frac{3\mu Ms}{{4\pi r^{4} }}$$

According to Eqs. (3) and (4), the unit bearing vector can be calculated as

5 $${\mathbf{r}}_{{\mathbf{0}}} = - \frac{{\mathbf{G}}}{{\left| {\mathbf{G}} \right|}}$$

According to Eqs. (2) and (4), the magnitude of bearing vector can be obtained

6 $$r = 3\frac{{\left| {\mathbf{B}} \right|}}{{\left| {\mathbf{G}} \right|}}$$

Hence, the bearing vector can be expressed as

7 $${\mathbf{r}} = r \cdot {\mathbf{r}}_{{\mathbf{0}}} = - \frac{{3\left| {\mathbf{B}} \right|}}{{\left| {\mathbf{G}} \right|^{2} }}{\mathbf{G}}$$

Equation (7) shows that the bearing vector can be calculated by total field |**B**| and its gradients data **G**. |**B**| and **G** both can be measured by a scalar magnetometer array. However, in fact, the parameter *s* is not a spatial constant. Under such conditions, the gradient of |**B**| is deduced as

8 $${\mathbf{G}} = \nabla \left| {\mathbf{B}} \right| = \frac{3\mu Ms}{{4\pi r^{4} }}[k_{2} {\mathbf{r}}_{{\mathbf{0}}} - k_{1} {\mathbf{m}}_{{\mathbf{0}}} ]$$

where *k*_1_ = *t*/*s*^2^, *k*_2_ = *t*^2^/*s*^2^ + 1. Then, the magnitude of **G** also can be obtained

9 $$\left| {\mathbf{G}} \right| = \frac{3\mu Ms}{{4\pi r^{4} }}k_{3}$$

where *k*_3_ = [1 + (1 − *t*^2^)*t*^2^/*s*^4^]^1/2^. In order to obtain the unit bearing vector **r**_**0**_, we define the unit vector of **G** from Eqs. (8) and (9).

10 $${\mathbf{G}}_{{\mathbf{0}}} = \frac{{\mathbf{G}}}{{\left| {\mathbf{G}} \right|}} = \frac{{k_{2} {\mathbf{r}}_{{\mathbf{0}}} - k_{1} {\mathbf{m}}_{{\mathbf{0}}} }}{{k_{3} }}$$

Hence, the unit bearing vector **r**_**0**_ can be deduced from Eq. (10)

11 $${\mathbf{r}}_{{\mathbf{0}}} = \frac{{k_{1} {\mathbf{m}}_{{\mathbf{0}}} + {\mathbf{G}}_{{\mathbf{0}}} k_{3} }}{{k_{2} }}$$

The magnitude of bearing vector can be obtained from Eqs. (2) and (9)

12 $$r = \frac{{\left| {\mathbf{B}} \right|}}{{\left| {\mathbf{G}} \right|}}k_{3}$$

The bearing vector can be expressed as

13 $${\mathbf{r}} = r.{\mathbf{r}}_{{\mathbf{0}}} = \frac{{k_{3} \left| {\mathbf{B}} \right|}}{{\left| {\mathbf{G}} \right|}}\frac{{k_{1} {\mathbf{m}}_{{\mathbf{0}}} + {\mathbf{G}}_{{\mathbf{0}}} k_{3} }}{{k_{2} }}$$

### Iteration algorithm

As can be seen in Eq. (13), bearing vector is determined by *k*_1_, *k*_2_, *k*_3_, total field |**B**| and its gradients data **G**. If we know **m**_**0**_ and **r**_**0**_, *k*_1_, *k*_2_ and *k*_3_ can be calculated by their definition, total field |**B**| and its gradients data **G** can be measured by a scalar magnetometer array. Generally, the magnetic target’s magnetic field is compounded by hard and induced magnetic field^21^. When the hard magnetic field is small and the induced magnetic field is the main component, the orientation of the target’s magnetic moment is parallel to the orientation of the geomagnetic field. In practice, the induced magnetic field is much larger than the hard magnetic field^18^. In this study, the induced magnetic field is only considered. Thus, the unit magnetic moment vector **m**_**0**_ can be calculated according to the magnetic inclination and declination, **m**_**0**_ can be expresssed as follows

14 $${\mathbf{m}}_{{\mathbf{0}}} = [\cos (I)\cos (D),\cos (I)\sin (D),\sin (I)]$$

where, *I* and *D* represent the magnetic inclination and declination of the local geomagnetic field at the measurement position, respectively. The values of them can be obtained from the international geomagnetic reference field (IGRF).

**r**_**0**_ and **m**_**0**_ can be calculated by Eqs. (5) and (14), respectively, as a result, *k*_1_, *k*_2_ and *k*_3_ can be calculated. However, the **r**_**0**_ calculated by Eq. (5) is approximation because parameter *s* is assumed to be a spatial constant. In order to improve the accuracy of bearing vector, an iteration algorithm is employed to update the **r**_**0**_ and the pseudocode of the iteration algorithm is expressed in Table 1.

**Table 1:**
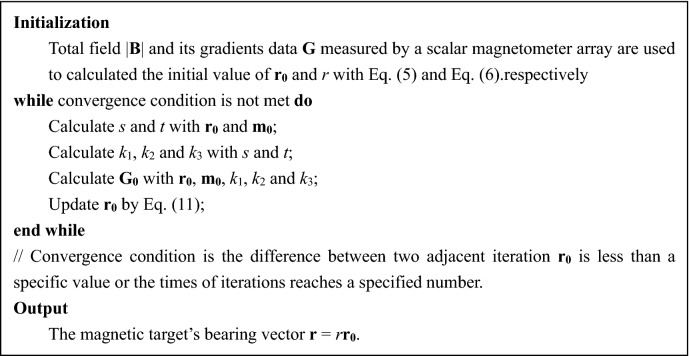
The pseudocode of the iteration algorithm.

## Methods

### Construction of the total field gradiometers and localization of the magnetic target

**Figure 1:**
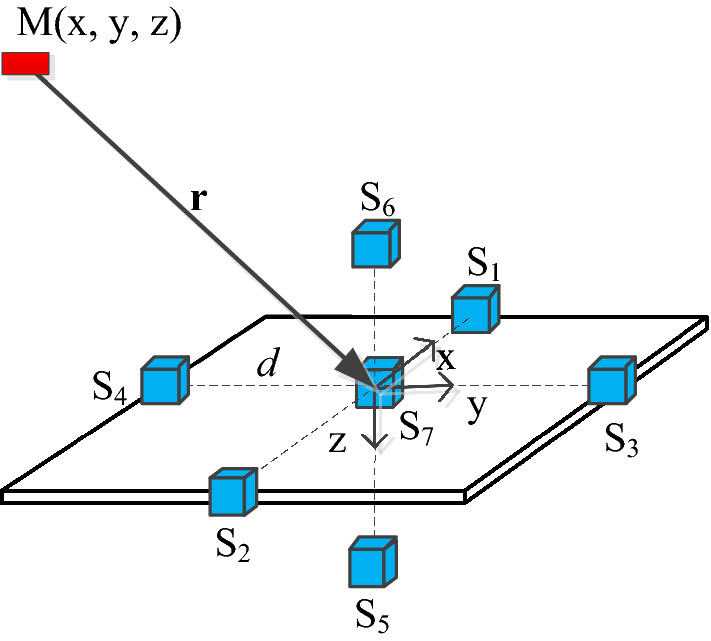
Schematic setup for simulation. The small cubes are scalar magnetometers.

To verify such a method, a proof-concept simulation is implemented. We proposed a structure of total field gradiometers, which includes seven scalar magnetometers named as S_*i*_ (*i* = 1–7), as shown in Fig. 1.

M(x, y, z) is the magnetic target. S_7_ is placed at the origin of a Cartesian coordinate system. The distance between magnetometers and origin is *d* = 0.5 m. The total field measured by S_*i*_ is |**B**|_*i*_ (*i* = 1–7). The gradient of total filed can be approximatively calculated as

15 $${\mathbf{G}} \approx [(\left| {\mathbf{B}} \right|_{1} - \left| {\mathbf{B}} \right|_{2} )/2d,(\left| {\mathbf{B}} \right|_{3} - \left| {\mathbf{B}} \right|_{4} )/2d,(\left| {\mathbf{B}} \right|_{5} - \left| {\mathbf{B}} \right|_{6} )/2d]$$

The initial value of **r**_**0**_ and *r* can be obtained as follows

16 $$\begin{gathered} r = \frac{{3\left| {\rm B} \right|_{7} }}{{\left| {\mathbf{G}} \right|}} \hfill \\ {\mathbf{r}}_{{\mathbf{0}}} = - \frac{{\mathbf{G}}}{{\left| {\mathbf{G}} \right|}} \hfill \\ \end{gathered}$$

**Figure 2:**
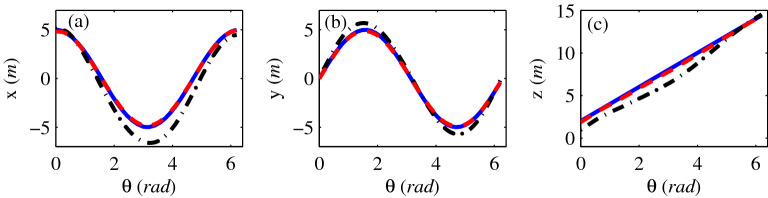
The estimated positions of (**a**) x-axis, (**b**) y-axis and (**c**) z-axis.

The magnitude of target’s magnetic moment is set 600 Am^2^. The magnetic inclination and declination is 63 and − 10°, **m**_**0**_ can be calculated as [0.4471, − 0.0788, 0.8910], the corresponding magnetic moment vector is [268.2560, − 47.3008, 534.6039] Am^2^. The movement path of target is defined in the following

17 $$\left\{ {\begin{array}{l} {x = 5\cos (\theta )} \\ {y = 5\sin (\theta )} \\ {z = 2 + 2\theta } \\ \end{array} } \right.$$

where the azimuth angle θ ∈ [0, 2π). The position of the magnetic target was estimated with and without iteration, the results are shown in Fig. 2.The dashdot line (black) in Fig. 2a–c is the estimated positions without iteration and the dashed line (red) is the estimated positions with iteration. For comparison purpose, the ideal value of the target’s positions (solid, blue) is also shown in Fig. 2a–c. As can be seen in Fig. 2, the estimated position accuracy without iteration is much lower than the proposed iterative method and there is a good agreement between the obtained positions with iterative method and the ideal value.

### Performance analysis

**Figure 3:**
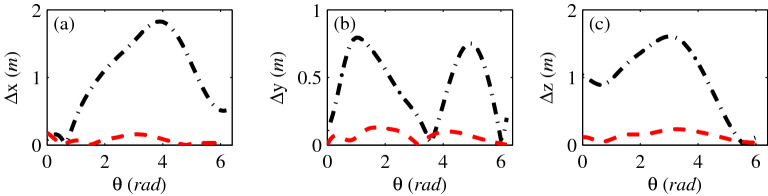
The curve of Δx (**a**), Δy (**b**), and Δz (**c**) versus *θ.*

In order to make a feasible quantitative analysis, we have defined the Δx, Δy, and Δz which denote the absolute value of the difference between the ideal positions and the estimated positions according x-axis,y-axis, and z-axis, respectively. The curves of Δx, Δy, and Δz versus *θ* are shown in Fig. 3. The dashdot line (black) is without iteration and the dashed line (red) is with iteration. It can be seen in Fig. 3 that the localization accuracy is greatly improved by the iterative method. The maximum of Δx without and with iterative method is 1.83 m and 0.18 m, respectively, i.e., the localization accuracy is improved by up to 10 times by using the iterative method according x-axis. In the same way, the localization accuracy is improved by up to 6 times and 7 times with iterative method according y-axis and z-axis, respectively.

**Figure 4:**
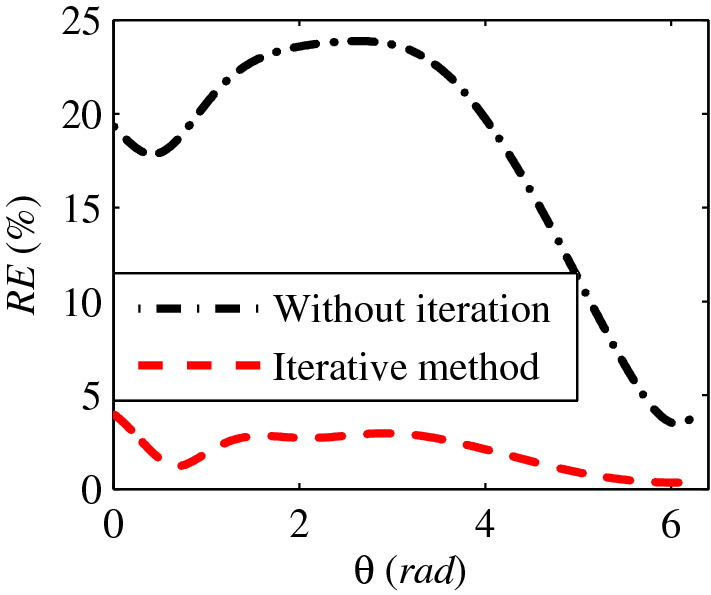
The *RE* versus *θ.*

Furthermore, the relative error (*RE*), which can be used to evaluate the accuracy of the estimated bearing vector similarity with respect to the ideal bearing vector, was defined as follows

18 $$RE = \frac{{\left| {{\mathbf{r}}_{{{\mathbf{est}}}} - {\mathbf{r}}_{{{\mathbf{ideal}}}} } \right|}}{{\left| {{\mathbf{r}}_{{{\mathbf{ideal}}}} } \right|}} \times 100\%$$

where **r**_**est**_ presents the estimated bearing vector, whereas **r**_**ideal**_ is the ideal (i.e. theoretical) bearing vector. RE versus *θ* is presented in Fig. 4. It is shown that the accuracy of the proposed iterative method was much higher than that of without iteration. The *RE* without iterative was even more than 20%, however, the *RE* with iterative method was kept below the level of 5%. If we assume that the *RE* is acceptable when it is less than 5%, then, the estimated bearing vector by the proposed method can be seen as the true position of the target with respect of any *θ*.

**Figure 5:**
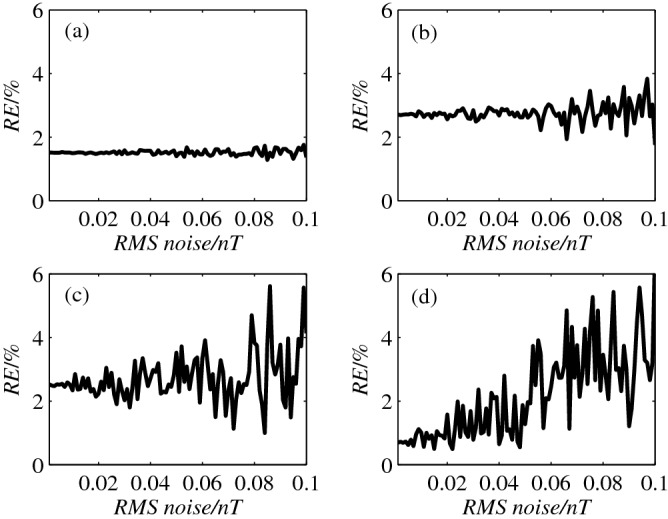
The *RE* versus RMS noise with *θ* of 30 (**a**), 120 (**b**), 210 (**c**) and 300 (**d**) degree.

In practice, the noise of the scalar magnetometers has an important impact on the measurement errors. In this simulation experiment, white Gaussian noise was added into the magnetometers. The target’s position is fixed with *θ* = 30, 120, 210 and 300°. The *RE* calculated with the iterative method with respect to the RMS noise is shown in Fig. 5. From Fig. 5a,b, we can see that the curves have a samll fluctuation with *θ* of 30 and 120°, which indicates that the noise has a little effect on the location accuracy. When *θ* is 210 and 300°, with the increase of RMS noise from 0.001 *nT* to 0.1 *nT*, the curves have a large fluctuations. For example, the *RE* of the bearing vector increases from 0.8% to 6% with *θ* is 300°. However, in most case, the *RE* is kept below of the level of 5%, hence, the proposed method has a high tolerance to the noise of the magnetometers.

**Figure 6:**
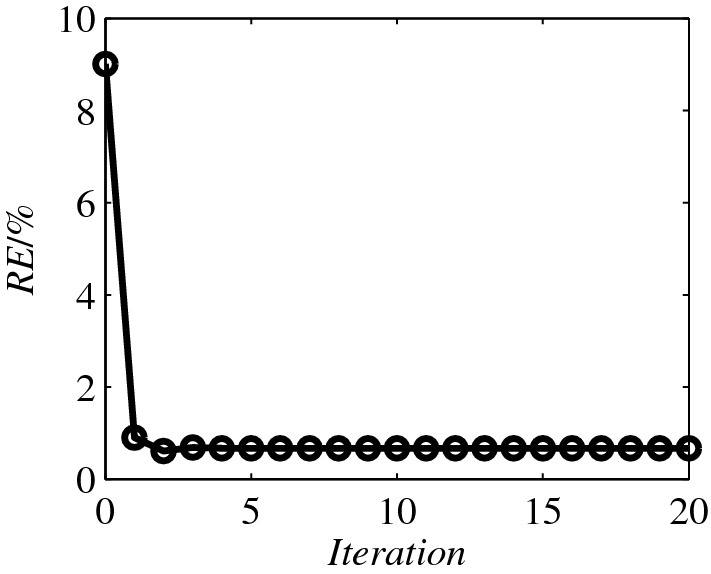
The *RE* due to iteration number.

For completeness, we also estimated the convergence of the proposed iteration algorithm. When the target’s position is fixed with *θ* = 300°, *RE* due to iteration number is shown in Fig. 6. From Fig. 6, we can see that the initial value of *RE* is 9.01% and it is relatively large, when iteration number is less than 4, the curve of *RE* has some fluctuation, howerver when iteration number is more than 5, the fluctuation is very tiny and the curve of RE is remained stable. It indicates that the difference between adjacent iteration of bearing vector is very small and the proposed method has an excellent performance in convergence.

## Discussion

In summary, a method for estimating magnetic target location by employing total field and its gradients data is proposed in this work. In the method, a scalar magnetometer array is constructed for the measurement of the total field and its gradients data. The approximate bearing vector can be calculated with the measurement data. In order to improve the localization accuracy, an iterative method is proposed and verified by simulation. The results show that the proposed method has a high accuracy of the magnetic target’s localization and a high tolerance to the measurement noise of the magnetometers.

## Data Availability

Data are available upon request from the corresponding author.
